# Efficient Shielding of Polyplexes Using Heterotelechelic Polysarcosines

**DOI:** 10.3390/polym10060689

**Published:** 2018-06-20

**Authors:** Philipp Michael Klein, Kristina Klinker, Wei Zhang, Sarah Kern, Eva Kessel, Ernst Wagner, Matthias Barz

**Affiliations:** 1 Department of Pharmacy, Ludwig-Maximilians-Universität (LMU) Munich, Pharmaceutical Biotechnology, Butenandtstrasse 5-13, D-81377 Munich, Germany; wei.zhang@cup.uni-muenchen.de (W.Z.); Sarah.Kern@cup.uni-muenchen.de (S.K.); ekessel86@gmail.com (E.K.); ernst.wagner@cup.uni-muenchen.de (E.W.); 2 Institute of Organic Chemistry, Johannes Gutenberg University, Duesbergweg 10-14, D-55128 Mainz, Germany; kklinker@uni-mainz.de; 3 Graduate School Materials Science in Mainz, Staudinger Weg 9, 55128 Mainz, Germany; 4 Nanosystems Initiative Munich, Schellingstraße 4, D-80799 Munich, Germany

**Keywords:** shielding agent, polysarcosine, biodistribution, click-chemistry, lipopolyplex, nucleic acid carrier

## Abstract

Shielding agents are commonly used to shield polyelectrolyte complexes, e.g., polyplexes, from agglomeration and precipitation in complex media like blood, and thus enhance their in vivo circulation times. Since up to now primarily poly(ethylene glycol) (PEG) has been investigated to shield non-viral carriers for systemic delivery, we report on the use of polysarcosine (pSar) as a potential alternative for steric stabilization. A redox-sensitive, cationizable lipo-oligomer structure (containing two cholanic acids attached via a bioreducible disulfide linker to an oligoaminoamide backbone in T-shape configuration) was equipped with azide-functionality by solid phase supported synthesis. After mixing with small interfering RNA (siRNA), lipopolyplexes formed spontaneously and were further surface-functionalized with polysarcosines. Polysarcosine was synthesized by living controlled ring-opening polymerization using an azide-reactive dibenzo-aza-cyclooctyne-amine as an initiator. The shielding ability of the resulting formulations was investigated with biophysical assays and by near-infrared fluorescence bioimaging in mice. The modification of ~100 nm lipopolyplexes was only slightly increased upon functionalization. Cellular uptake into cells was strongly reduced by the pSar shielding. Moreover, polysarcosine-shielded polyplexes showed enhanced blood circulation times in bioimaging studies compared to unshielded polyplexes and similar to PEG-shielded polyplexes. Therefore, polysarcosine is a promising alternative for the shielding of non-viral, lipo-cationic polyplexes.

## 1. Introduction

Therapeutic nucleic acids are powerful tools, which can be used to specifically control gene expression inside cells [1,2,3,4,5]. For several diseases, including severe metastatic tumors, systemic delivery is required to achieve therapeutic effects. Naked oligonucleotides have limited stability in biological fluids because they are actively targeted and consequently, degraded by nucleases. Although this issue might be addressed by chemical modifications [6,7], the renal clearance of small oligonucleotides or siRNA usually occurs within a few minutes, which limits the time to reach their desired site of action [8,9]. Lipopolymer micelles, liposome-based formulations, and polymer-based complexes increase the size usually beyond the renal cut-off and thus enhance circulation times, whenever a stealth-like corona protects the systems from unspecific aggregation [6,10,11,12,13,14,15,16,17,18,19,20,21]. Various cationic lipids, polycations and combinations with helper lipids [4,14,16,17,22,23,24] were used to form lipoplexes or lipid nanoparticles (LNPs) [1,11,25,26,27,28,29,30]. Precise editing of the compounds’ chemical structure enables the fine-tuning of a carrier’s stability and size, but also other properties, which are important for the delivery process like cellular uptake, endosomal escape ability, and cell tolerability [31,32,33,34]. 

Solid phase-supported synthesis (SPS) is a very convenient and precise way to optimize a delivery system in a step-wise manner [18,35]. Recently, we developed our own customized amino acids, such as succinoyl tetraethylene pentamine (Stp), which contains of short defined repeats of the ethylenediamine motif prepared in boc/fmoc protected form. With artificial building blocks, natural α-amino acids and fatty acids, we sequentially synthesized monodisperse cationic oligomers via SPS, which are highly adaptable to different demands in the field of gene delivery [8,12,18,31,32,33,36,37,38,39]. By precise incorporation of a bioreducible cleavage site between the cationic and a lipophilic block, for instance, it was possible to destabilize polyplexes only after reaching the cytoplasm of the cell [39]. Thereby the carrier system remained stable in serum and transfection efficiency as well as cell viability could be increased in certain cell lines. 

Besides size and stability, the surface character of a nanoparticle is of utmost importance for its systemic delivery. Shielding agents attached to the surface prevent interactions with neighboring particles and/or blood components, which usually leads to extended circulation in the body’s bloodstream [40,41,42]. Already in 1990, it could be demonstrated that polyethylene glycol (PEG) could extend the blood circulation half-life of systemically injected liposomes from <30 min to several hours [40]. Its hydrophilic character enables PEG to generate a hydrated shell covering the nanoparticles and thereby sterically reduce unwanted interactions with biomolecules or other poly- or lipoplexes [43]. PEG is the most prominent shielding agent and has often been used to shield cationic polyplexes in numerous applications [44,45,46,47,48,49]. A major drawback, however, is that more and more researchers in academia or industry observe immune responses towards PEGylated nanoparticles [43,50,51,52,53,54,55]. For this reason, several new alternatives were evaluated for shielding, such as natural proteins [56], oligosaccharides [57,58], poly(*N*-(2-hydroxypropyl)methacrylamide) (pHPMA) [58,59,60], hydroxyethyl starch (HES) [61] or polypeptides (poly(glutamic acid) [62], poly(hydroxyethyl-l-asparagine) [63], poly(hydroxyethyl-l-glutamine) [63], proline-alanine-serine motif (PAS) [64,65]). Nevertheless, according to the Whitesides’ rules for protein resistant surfaces an ideal alternative to PEG should mimic its chemical properties, being a hydrophilic, non-charged polymer and a weak hydrogen acceptor without donor properties, which is not the case for all above-mentioned polymers. In contrast, polysarcosine fulfills all the described criteria and has already demonstrated protein resistant properties on various surfaces [66,67,68]. In addition, it can be also synthesized by living controlled ring-opening polymerization of the corresponding *N*-carboxyanhydrides (NCAs) [69,70]. However, in vivo data on polysarcosine is rarely reported in literature [71]. In contrast to polypeptides, the side chain of polypeptoids is situated at the nitrogen rather than the α-carbon, in the case of pSar the nitrogen is methylated. As a result, polysarcosine adopts a random coil conformation in aqueous solution and possesses a comparable second virial coefficient and molecular weight dependency like PEG [72]. All these properties mediate high resistance against protein adsorption [73] and make it in theory an ideal material for shielding electrostatic complexes in vivo [74]. Importantly, it has been reported that polysarcosine has so far demonstrated neglectable complement activation or immunogenicity in mouse, rat and rabbit animal models [75,76]. pSar-shielded polyplexes, micelles, colloids and nanohydrogels demonstrated the absence of aggregation in human serum [77,78,79,80].

In the current work, we have incorporated azide domains into a previously described redox-sensitive T-shaped bis-(cholanic acid amido) oligoaminoamide siRNA carrier system [39] and used strain-promoted azide-alkyne cycloaddition (SPAAC) reaction to equip the lipopolyplexes’ surface with ~8 kDa polysarcosine (DP = 119) chains. We report on the ability of polysarcosine to shield siRNA lipoplexes and analyzed the in vivo stability and biodistribution after intravenous administration into mice. In a second approach, we modified the system with a folate ligand to target the folate receptor overexpressed on certain cancer cells [81,82,83,84,85,86].

## 2. Materials and Methods

### 2.1. Materials

Protected Fmoc-α-amino acids, *N*,*N*-dimethylformamide (DMF), *N*,*N*-diisopropylethylamine (DIPEA), trifluoroacetic acid (TFA) and 2-chlorotrityl chloride resin for solid-phase syntheses were purchased from Iris Biotech (Marktredewitz, Germany). 1-hydroxybenzotriazole (HOBt), triisopropylsilane (TIS), 5β cholanic acid. Dimethylformamide (DMF, 99.8% Extra) for Dibenzoazacyclooctyneamine polysarcosine (DBCO-pSar) syntheses was purchased from Acros Organics (Nidderau, Germany), further dried over CaH2 and fractionally distilled in vacuo. Tetrahydrofuran (THF) and folic acid (FolA, 96–102% pure) was purchased from Acros Organics. Triethylamine (TEA) and *N*,*N*-diisopropylethylamine (DIPEA) were dried over NaOH and fractionally distilled in vacuo. (Benzotriazol-1-yloxy)tripyrrolidinophosphonium hexafluoro-phosphate (PyBOP), 2-(1*H*-benzotriazol-1-yl)-1,1,3,3-tetramethyluronium hexafluorphosphate (HBTU) and microreactors were purchased from MultiSynTech (Witten, Germany). Cell culture media, antibiotics and fetal bovine serum (FBS) were obtained from Invitrogen (Karlsruhe, Germany), HEPES from Biomol GmbH (Hamburg, Germany), agarose (NEEO Ultra-quality) from Carl Roth GmbH (Karlsruhe, Germany), glucose from Merck (Darmstadt, Germany), and GelRed™ from VWR (Darmstadt, Germany). d-Luciferin sodium salt and cell culture 5 × lysis buffer were purchased from Promega (Mannheim, Germany). Ready-to-use siRNA duplexes were purchased from Axolabs GmbH (Kulmbach, Germany): eGFP-targeting siRNA (siGFP) (sense: 5′-AuAucAuGGccGAcAA GcAdTsdT-3′; antisense: 5′-UGCUUGUCGGCcAUGAuAUdTsdT-3′) for silencing of eGFPLuc; control siRNA (siCtrl) (sense: 5′-AuGuAuuGGccuGuAuuAGdTsdT-3′; antisense: 5′-CuAAuAc AGGCcAAuAcAUdTsdT-3′); Cy5-labled siRNA (Cy5-siAHA1) (sense: 5′-(Cy5)(NHC6)GGAu GAAGuGGAGAuuAGudTsdT-3′; antisense: 5′-ACuAAUCUCcACUUcA- UCCdTsdT-3′); Cy7-labled siRNA (Cy7-siAHA1) (sense: 5′-(Cy7)(NHC6)GGAuGAAGuGGAG- AuuAGudTsdT-3′; antisense: 5′-ACuAAUCUCcACUUcAUCCdTsdT-3′) small letters: 2’methoxylated; s: phosphorothioate. All other chemicals were obtained from Sigma (Munich, Germany), Merck, Iris Biotech (Marktredwitz, Germany) or AppliChem (Darmstadt, Germany), Acros Organics, Fluka (Munich, Germany) or Alfa Aesar (Karlsruhe, Germany).

### 2.2. Synthesis of Oligomers and DBCO Shielding Agents

#### 2.2.1. Synthesis of Oligomers

See Supporting Information for detailed descriptions of the syntheses of oligomers.

#### 2.2.2. Synthesis of Sarcosine-*N*-Carboxyanhydride 

The synthesis was performed as described in Klinker et al. [71] Sarcosine (15.16 g, 170.2 mmol, 1 eq) was weighed into a pre-dried three-necked flask and dried under vacuum for one hour. Absolute (abs) tetrahydro furane (THF) (300 mL) was added under a steady nitrogen flow. Two gas washing bottles with aqueous sodium hydroxide solution were connected to the apparatus. Diphosgene (16.26 mL, 134 mmol, 0.8 eq) was added dropwise via syringe. The colorless suspension was heated to 70 °C resulting in a clear solution after three hours of stirring. The solvent was evaporated under reduced pressure resulting in a brown oil as crude reaction product. The obtained oil was heated to 50 °C and dried under reduced pressure yielding an amorphous solid. The solid was redissolved in 60 mL THF and precipitated by adding 300 mL abs n-hexane. The precipitate was filtered off under nitrogen-atmosphere and dried with a stream of dry N_2_ for 60–90 min to remove remaining traces of solvents. The next day, the solid was dried in high vacuum for 2 h in the sublimation apparatus followed by sublimation at 80–85 °C and <1 × 10^−2^ mbar. The product was collected from the sublimation apparatus in a glove box the same day. Colorless crystals were obtained (50–67%). mp = 104.3 °C; ^1^H-NMR (300 MHz, CDCl_3_) δ [ppm] = 2.86 (s, 3H, N−CH_3_), 4.22 (s, 2H, N−CH_2_CO).

#### 2.2.3. Synthesis of DBCO-pSar

DBCO-pSar was synthesized using ring-opening polymerization of SarNCA as described in Klinker et al. [71] In a typical experiment, SarNCA (461.8 mg, 4.012 mmol) were transferred under N_2_ counter flow into a pre-dried Schlenk-tube, equipped with a stir bar and dried again in vacuum for 30 min. The NCA was dissolved in dry DMF (3.5 mL). A stock solution of DBCO-amine (0.074 mmol, 1/110 eq, M/I = 110) in DMF (2 mL) was freshly prepared and 1 mL of this stock solution was added to the monomer solution with syringe. The solution was stirred at a temperature of 40 °C and kept at a constant pressure of 1.25 bar of dry N_2_ via the Schlenk-line to prevent impurities from entering into the reaction vessel while allowing CO_2_ to escape. Completion of the reaction was confirmed by infrared spectroscopy (disappearance of the NCA peaks (1853 and 1786 cm^−1^)). Directly after completion, the polymer was precipitated in ice-cold diethyl ether and centrifuged (4500 rpm at 4 °C for 15 min). After discarding the liquid fraction, new diethyl ether was added and the polymer was resuspended via sonication. Again, the suspension was centrifuged and the procedure was repeated. The polymer was subsequently dissolved in water and lyophilized to obtain a fluffy powder (279.7 mg, 98%). ^1^H-NMR: (400 MHz, DMSO−d_6_): δ [ppm] = 0.88–0.79 (m, ini, 9H, –C(CH_3_)_3_), 2.58–3.11 (br, 3nH, N–CH_3_), 3.73–4.57 (br, 2nH, –CO–CH_2_–N), = 7.86–7.10 (m, 8H, benzylic protons).

#### 2.2.4. Synthesis of DBCO-pSar-Ac

*DBCO-pSar_119_* (Mn = 8735 g mol^−1^) (36 mg, 0.004 mmol), acetic anhydride (4.2 mg, 3.9 µL, 0.04 mmol), and triethylamine (10.7 mg, 14.6 µL, 0.08 mmol) were dissolved in absolute DMF (1 mL) and stirred at 25 °C for 24 h under an argon atmosphere. Subsequently, the polymer was precipitated in diethyl ether, extensively dialyzed against water (MWCO = 3500 g mol^−1^), and lyophilized. Yield after dialysis: 25 mg (69%).

#### 2.2.5. Synthesis of DBCO-pSar-FolA

DBCO-pSar_110_ (Mn = 8095 g mol^−1^) (55.8 mg, 0.007 mmol) was separately dissolved in absolute 5mL dimethylsulfoxid (DMSO). Folic acid (30.4 mg, 0.068 mmol), HBTU (26.1 mg, 0.068 mmol), and HOBt (9.31 mg, 0.068 mmol) were dissolved in 5mL DMSO and cooled to 0 °C. DIPEA (17.8 mg, 24.0 µL, 0.138 mmol) was added and the mixture was left to react for 30 min at 0 °C. The in situ formed activated ester was added to the predissolved polymer and the reaction mixture was stirred at 25 °C for 24 h under an argon atmosphere. The crude reaction product was purified by size exclusion chromatography in DMSO using a Sephadex LH-20-packed column. The purified conjugate was lyophilized from water. Yield after SEC: 31 mg (55%).

#### 2.2.6. Gel Permeation Chromatography

Polymer molecular weight and dispersity index were determined using gel permeation chromatography (GPC). GPC in hexafluoro-2-propanol (HFIP) was performed with 3 g L^−1^ potassium trifluoroacetate (KTFA) at a temperature of 40 °C. The columns were packed with modified silica (PFG columns, particle size: 7 µm; porosity: 100 and 1000 Å). A UV/vis detector (UV-2075 Plus, JASCO, Groß-Umstadt, Germany, λ = 230 nm; λ = 330 nm for folic acid detection) and a refractive index detector (G 1362A RID, Jasco) were used to detect the polymer. Molecular weights were calculated by using calibration performed with PMMA standards (Polymer Standards Services GmbH, Mainz, Germany) Toluene was used as the internal standard.

#### 2.2.7. UV-Vis Spectroscopy

UV-vis absorbance spectra were recorded using a V-630 spectrophotometer (Jasco) with water being the solvent.

### 2.3. Formation of siRNA Polyplexes

siRNA was diluted with 20 mM HEPES buffered 5% glucose pH 7.4 (HBG) to a concentration of 50 ng/µL for in vitro experiments and 500 ng/µL for in vivo experiments. According to the indicated nitrogen/phosphate (*N*/*P*) ratio, the oligomer solution was prepared in a separate tube. Only protonatable nitrogens of an oligomer were considered in the *N*/*P* calculation. The same volume of siRNA solution was added to the oligomer. The mixture was rapidly pipetted at least five times and incubated for 40 min at RT yielding a polyplex solution with 25 or 250 ng of siRNA/µL respectively.

### 2.4. Functionalization of Polyplexes with DBCO Reagents

For functionalization of siRNA polyplexes with DBCO click agents, solutions with DBCO reagents were prepared in ¼ of the volume of the previously prepared polyplex solutions. The concentration of the solution was calculated according to the indicated equivalents (eq) used. Equivalents represent the molar ratio of shielding agent to cationic oligomer in the polyplex solution. The reaction time was 16 h for biophysical and in vitro assays and 4 h for in vivo experiment respectively.

### 2.5. siRNA Binding Assays

siRNA binding assays were performed analogously as described in Klein et al. [86]. A 1% agarose gel was prepared by dissolving agarose in TBE buffer (10.8 g of Trizma base, 5.5 g of boric acid, 0.75 g of disodium EDTA, and 1 L of water) and subsequent boiling. After cooling down to about 50 °C, GelRed^TM^ was added. Formulations were prepared with 500 ng of siRNA per sample. Samples were placed into the pockets after 4 µL of loading buffer (prepared from 6 mL of glycerine, 1.2 mL of 0.5 M EDTA, 2.8 mL of H_2_O, 0.02 g of bromophenol blue) was added. Electrophoresis was performed at 70 V for 60 min.

### 2.6. Particle Size and Zeta Potential Measurements

Polyplexes were formed using 2 µg siRNA in a total volume of 80 µL. Dynamic light scattering (DLS) measurements of polyplex solutions were performed in a folded capillary cell (DTS 1070). A Zetasizer Nano ZS with backscatter detection (Malvern Instruments, Worcestershire, UK) was used for the measurements. For size measurements, the equilibration time was 0 min, the temperature was set to 25 °C and an automatic adjustment of the attenuator was used. The refractive index of the solvent was set to 1.330 and the viscosity was set to 0.8872 mPa·s. Each sample was measured three times. For detection of the zeta potential, the sample was diluted to 800 µL volume with 10 mM NaCl solution. Measurements with at least 6 runs were performed. Zeta potentials were calculated using the Smoluchowski equation. 10 to 15 sub runs each lasting 10 s (n = 3) were measured.

### 2.7. Cell Culture

The mouse neuroblastoma cells (Neuro2a) were cultured in Dulbecco’s Modified Eagle Medium (DMEM) low glucose medium (Sigma). As FR-expressing cell lines, human cervix carcinoma cells (KB cells), and human cervix carcinoma cells transfected stably with the eGFPLuc (enhanced green fluorescent protein/luciferase) gene (KB/eGFPLuc cells) were cultured in folate-free Roswell Park Memorial Institute medium (RPMI) 1640 (Invitrogen). All media were supplemented with 10% FBS, 100 U/mL penicillin, and 100 μg/mL streptomycin. The cells were cultured in ventilated flasks in the cell incubator at 37 °C and 5% CO_2_ in a humidified atmosphere. After a confluency of 80–90% was reached, cells were harvested.

### 2.8. Cell Association and Internalization of siRNA Polyplexes Measured with Flow Cytometry

For untargeted polyplexes, Neuro2a cells were seeded in 24-well plates with 5 × 10^4^ cells/well 24 h prior the experiment, and fresh growth medium was provided immediately before the experiment. Polyplexes containing 1.5 µg of siRNA (20% Cy5-labeled siRNA + 80% unlabeled siRNA) were pipetted into each well and incubated for four hours at 37 °C in 5% CO_2_. Polyplexes non-specifically associated to the cell surface were then removed by incubating cells with 500 I.U. heparin. For folate-targeted polyplexes, KB cells were seeded in 24-well plates with 5 × 10^4^ cells perwell at 24 h before the experiment, and fresh growth medium was provided before the experiment. Polyplexes containing 1.5 µg of siRNA (20% Cy5-labeled siRNA + 80% unlabeled siRNA) were pipetted into each well, incubated 30 min on ice for cell association or 45 min at 37 °C in 5% CO_2_ in case of cellular internalization. Cells were then washed with PBS to remove free polyplexes. In case of testing the cellular internalization, polyplexes non-specifically associated to the cell surface were then removed by incubation with 500 I.U. heparin. Next, cells were collected and resuspended in PBS buffer containing 10% of FBS. All samples were analyzed by flow cytometry with CyanTM ADP (Dako, Hamburg, Germany) using an excitation wavelength of 635 nm, and detecting the emission at 665 nm. Dead cells were differentiated by fluorescence of DAPI and removed by gating in order to only analyze cellular uptake of polyplexes in living cells. Flow cytometry data were analyzed using FlowJo 7.6.5 flow cytometric analysis software by FlowJo, LLC (Ashland, OR, USA).

### 2.9. Confocal Laser Scanning Microscopy (CLSM)

Neuro2a cells were seeded at a density of 3 × 10^4^ cells/well in 300 μL of growth medium into an 8-well Lab-Tek chamber slide (Nunc, Schwerte, Germany) 24 h prior to treatment. Polyplexes were formed as described using a 1.5 µg of a mixture of 80% of siCtrl and 20% Cy5-labeled siRNA and oligomer at *N*/*P* 12 in 60 μL of HBG followed by the indicated agent in 20 µL. Cells were incubated with 220 μL of fresh growth medium and the polyplex solution was applied. For the uptake study, the incubation with polyplexes was at 37 °C for 4 h. The growth medium was removed, cells were washed twice with 300 µL of PBS and fixed with 4% PFA solution for 30 min at RT. Cell nuclei were stained with DAPI. For image acquisition, a TCS SP8 confocal microscope (Leica, Mannheim, Germany) was used.

### 2.10. Mouse Tumor Model

Female six- to seven-week-old nude mice, Rj: NMRI-nu (nu/nu) (Janvier, Le Genest-Saint-Isle, France), were housed in isolated ventilated cages under pathogen-free condition with a 12 h light/dark interval and were acclimated for at least seven days prior to experiments. Water and food were provided *ad libitum*. Injection of animals was performed subcutaneously with 5 × 10^6^ Neuro2a cells. The tumor volume was measured by caliper and calculated as [0.5 × (longest diameter) × (shortest diameter)^2^] and the body weight was recorded. All animal experiments were performed according to the terms stated in the proposal “Entwicklung von Sequenz-definierten Oligomeren als Träger für die zielgerichtete Einbringung neuer molekularer Therapeutika in Tumore“ that was approved by the local animal ethics committee and the government of Oberbayern at the 26 May 2014. All animal studies were performed according to guidelines of the German Animal Welfare Act.

### 2.11. Biodistribution Study

A mixture of 50% unlabeled control siRNA (siCtrl) and 50% of Cy7-labeled siRNA (Cy7-siAHA1) in HBG was used for all near infrared (NIR) in vivo imaging experiments. Tumors of mice were allowed to grow until reaching a size of 500–1000 mm^3^. The mice (n = 2/per group) were anesthetized with 3% isoflurane in oxygen. siRNA polyplexes containing 50 µg siRNA (50% Cy7-labeled—*N*/*P* 10) in 250 µL (prepared from 100 µL of siRNA solution, 100 µL of oligomer solution and 50 µL of agent solution or buffer) or HBG as negative control were injected intravenously (i.v.), and fluorescence intensity was measured with a CCD camera at different time points. For evaluation of images, the intensity of fluorescence signals was analyzed after color bar scales were equalized using the IVIS Lumina system and the Living Image software 3.2 (Caliper Life Sciences, Hopkinton, MA, USA).

## 3. Results and Discussion

### 3.1. Design and Synthesis of Lipo-Oligomers for Click Chemistry

Previously, we have established a novel class of redox-sensitive lipo-oligomers that were prepared by solid-phase supported synthesis (SPSS) serving as carriers for siRNA delivery [39]. Beside all beneficial effects of lipid-based delivery systems, such as endosomal escape capability and enhanced nanoparticle stability, the in vivo distribution is often limited to certain tissues, primarily liver, lung and spleen [87,88,89]. In previous work, it has been shown that T-shape oligomers similar to the ones, which were synthesized in this approach demonstrate strongest retention in liver tissue [89,90]. This observation might be related to a high stickiness of cationic particles, but it is also possible that certain serum proteins, which incorporate onto nanoparticle surfaces may impair tissue specificity [88,91,92,93]. An efficient shielding should reduce both types of interactions and should enable a better distribution in the body. Therefore, one of the top candidates from redox-sensitive lipo-oligomers, T-shape structure ***T-0N_3_*** (published as ID ***992*** in [39]) was chosen and extended by click-reactive azide functionality. After the formation of siRNA lipopolyplexes, the surface was further modified with the shielding agents.

**Figure 1:**
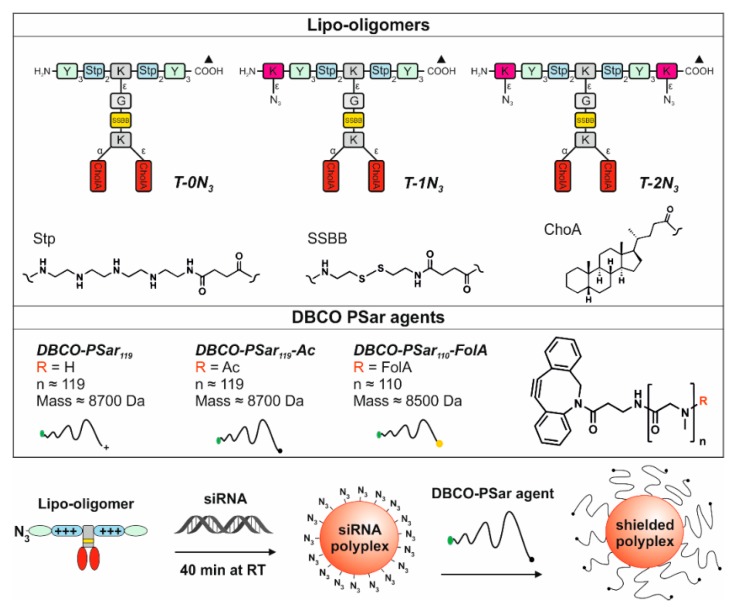
Overview of chemical compounds. Table, **top**: structural illustration of sequence-defined oligomers with T-shape topology; ***T-0N_3_*** (ID: ***992*** published [39]), ***T-1N_3_*** (ID: ***1073***) and ***T-2N_3_*** (ID: ***1086***) with no, one or two terminal azidolysines K(N3). Further units of the oligomers: Stp: succinoyl-tetraethylene-pentamine, ssbb: succinoyl-cystamine, CholA: 5β-Cholanic acid, Y: tyrosine, K: lysine, G: glycine. The broken lines represent amide linkages, the triangle (▲) is the starting point of the synthesis. IDs are unique database identification numbers. Table, **bottom**: chemical structure of the shielding agents ***DBCO-pSar_119_***, ***DBCO-pSar_119_-Ac*** and ***DBCO-pSar_110_-FolA***. Scheme of the formulation of a shielded polyplex.

***T-0N_3_*** was chosen as a lead structure and starting point for further modifications, because it forms stable siRNA polyplexes smaller than 200 nm in hydrodynamic diameter, which show high transfection efficiency in mouse neuroblastoma (Neuro2a) cells. This structure combines natural amino acids and artificial building blocks (Figure 1, top). It consists of four repeats of the artificial, cationic polyamino acid succinoyltetraethylenepentamine (Stp) used for complexation of nucleic acid and for endosomal buffering. Two trimeric tyrosine units flanking the cationic domain are able to stabilize the polyplex due to their hydrophobicity and π–π stacking ability. In between the cationic Stp units, two hydrophobic cholanic acids branch off the backbone (T-shape). 

This hydrophobic domain mediates efficient lipopolyplex stabilization. The lipid and cationic domains are connected with a bioreducible linking unit (ssbb) [39]. In this approach, the azide function was incorporated into the lipo-oligomer during standard Fmoc solid phase-supported synthesis via an azidolysine residue at the *N*- and/or *C*-terminus of the backbone (structures ***T-1N_3_*** with one azide and ***T-2N_3_*** with two azides, Figure 1, top). Consequently, the structure can be subsequently further modified with an alkyne-bearing functional group via click chemistry.

### 3.2. Synthesis of DBCO-Modified Polysarcosine

Polysarcosine is a hydrophilic, nonionic peptoidic structure with exclusively weak hydrogen bond acceptor properties. As shown empirically by Whitesides and co-workers for protein-resistant surfaces, these properties are essential to achieve “stealth”-like properties in a material [71,74]. Polysarcosine can be functionalized at its N-terminal (via post-polymerization modification) and C-terminal (via functional initiators) end. It is conveniently synthesized by controlled living ring-opening polymerization of α-amino acid *N*-carboxyanhydrides (NCA) with low dispersity index (Ð_GPC_ ≤ 1.1; see Table S1) [71]. Initiating the reaction with dibenzoazacyclooctyneamine (DBCO-amine) leads to a C-terminal DBCO end group (Figure S1A) [94]. To ensure end group accessibility and steric stabilization at once, we aimed for a degree of polymerization of around 115, which correlates with the PEG5k used as reference material. Therefore, a theoretical degree of polymerization of 115 was set by the monomer to initiator ratio. The polymerization was carried out under the conditions recently reported by Klinker et al. [95], NMR end group and SEC analysis (using pSar standards as described by Weber et al. [72]) revealed a number average degree of polymerization (DP) of 119 and a number average molecular weight of 8735 g mol^−1^ (Table S1), which is within the experimental error. The experimentally determined molecular weight is perfectly in line with the calculated one. The synthesized polymer displays a symmetrical SEC elugram indicating a Poisson-like molecular weight distribution, as expected for the amine initiated *N*-substituted glycine *N*-carboxyanhydride (NNCA) polymerization. The terminal DBCO can be clearly detected in the ^1^H-NMR spectra and thus can be employed for the strain promoted azide-alkyne cycloadditon (SPAAC) with azides. For SPAAC no catalyst is needed, no toxic by-products are generated, and no side reactions with other functional domains of the oligomer can occur [96]. Mixing azide-modified cationic lipo-oligomers with siRNA leads to spontaneous assembly of lipopolyplexes. Due to oligomer excess, several azide functionalities are accessible on the polyplex surface and can serve as attachment points for functionalization with DBCO-modified pSar. The N-terminal free amine group can further be modified with carboxylic acid-bearing molecules to introduce a second functionality, e.g., targeting ligands such as folic acid or alternatively may be capped by acetylation to remove the terminal amine, which is positively charged in aqueous solution of neutral pH. All synthesized agents are presented in Figure 1.

### 3.3. Polyplex Formation and pSar-Shielding

For polyplex formation, the structures ***T-1N_3_*** and ***T-2N_3_*** were incubated with siRNA for 40 min at a final concentration of 25 ng siRNA/µL. The nucleic acid binding ability was evaluated by measuring the electrophoretic mobility of incorporated siRNA within an agarose gel. Different *N*/*P* values depict the ratio of protonatable amines (*N*) within an oligomer to the phosphates (*P*) of the siRNA. Like its azide-free analogue ***T-0N_3_*** [39], the new azide-containing oligomers showed complete retention of the nucleic acid in the pockets of the gel at an *N*/*P* ratio of 12 (Figure S2). The binding ability observed for structures containing one or two azides did not differ.

Next, the heterotelechelic polysarcosine with DBCO-end group (***DBCO-pSar_119_***; Figure 1, bottom) was used to react with the azides on the preformed polyplexes (*N*/*P* ratio 12) to introduce a shielding layer. The SPAAC was allowed to proceed until full conversion for 16 h (see scheme in Figure 1, bottom).

**Figure 2:**
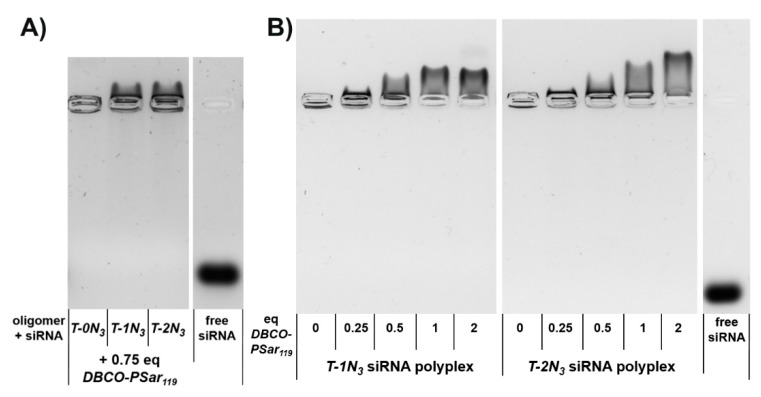
Electrophoretic mobility of siRNA lipopolyplex formulations analyzed with an agarose gel shift assay (**A**) siRNA polyplexes formed with lipo-oligomers bearing no (***T-0N_3_***), one (***T-1N_3_***) and two (***T-2N_3_***) azide functions incubated with 0.75 equivalents of ***DBCO-pSar_119_*** 1% agarose gel, 70 V, 80 min runtime; (**B**) Formulations with increasing equivalents (eq mol/mol) of ***DBCO-pSar_119_***. 0.75% agarose gel, 100 V, 80 min runtime. Polyplexes were incubated for 40 min at *N*/*P* 12, followed by ***DBCO-pSar_119_*** addition for 16 h at room temperature. The right lane in each gel shows the running distance of free siRNA not complexed by lipo-oligomers.

Afterwards, the influence of the pSar shielding agent on electrophoretic mobility was evaluated with respect to the number of azide functions incorporated into the polyplex-forming core structures (N_3_ = 0, 1, 2). The amount of ***DBCO-pSar_119_*** added to the polyplexes was kept constant (Figure 2A). In this experiment, only the azide-bearing polyplexes migrated in the agarose gel towards the cathode, whereas the azide-free polyplex remained in the loading pocket. This demonstrates that a covalent bond connecting the shielding agent to the nanoparticle is crucial to provide this migratory effect. The migration of the integrated nucleic acid against its own negative charge shows that it is fully shielded against the force of the electric field. With increasing equivalents of ***DBCO-pSar_119_***, stronger migration could be observed. This effect can be explained by the degree of polyplex surface modification (Figure 2B). For the oligomer with only one azide functionality (***T-1N_3_***), maximum migration was achieved with equimolar amounts of DBCO click agent (1 eq/oligomer). More ***DBCO-pSar_119_*** (2 eq) did not increase the effect. ***T-2N_3_*** siRNA polyplexes with two azide functionalities within the carrier also revealed the maximum migration for equimolar ratios of azide to DBCO (2 eq ***DBCO-pSar_119_***). These findings are in line with covalent modification with PEG5k [86].

A second indirect measure for the efficiency of polyplex shielding is the zeta potential or electrochemical mobility. The latter can be determined by measuring a particle’s mobility in an electric field with light scattering. 

In this respect, we observed that the positive zeta potential of an unshielded particle can be strongly reduced from 21 mV to 6 mV in case of ***T-1N_3_*** polyplexes and from 17 mV to 3 mV in case of ***T-2N_3_*** polyplexes, when the particle is shielded with an excess of ***DBCO-pSar_119_*** (Table 1). By using 0.5 eq ***DBCO-pSar_119_***/oligomer, the zeta potential can already be reduced to 50%. It should be noted here that due to the N-terminal cationic tail group, the zeta potential always remained slightly positive. As determined by single-angle dynamic light scattering (DLS), hydrodynamic diameters of the polyplexes were approximately 100 nm. With increasing amounts of ***DBCO-pSar_119_***, the nanoparticle size increased by up to 16 nm in diameter. The most plausible explanation for the increase in size seems to be pSar covering the particle surface.

### 3.4. Evaluation of pSar-Shielding Agents In Vitro

Through the incorporation of pSar the unspecific interaction of polyplexes with cell membranes should be efficiently reduced as already demonstrated for other stealth-like polymers, e.g., PEG. To prove our assumption we performed uptake studies with pSar-shielded polyplexes. Formulations were prepared with Cy5-labled siRNA for this assay to follow the fluorescent cargo, incubated with neuroblastoma Neuro2a cells for 4 h at standard culture conditions and analyzed by flow cytometry. The signal intensity of cells bearing fluorescent dyes correlates with the amount of polyplexes being internalized (Table 2 and Figure S3). Unshielded material and material shielded with low equivalents of ***DBCO-pSar_119_*** showed significant uptake into cells already after 4 h incubation time for polyplex formulations prepared with one or two azide-bearing backbones (***T-1N_3_*** and ***T-2N_3_***). For ***T-1N_3_*** formulations, a significant reduction in fluorescence intensity of more than 50% was observed for 1 eq of ***DBCO-pSar_119_*** per oligomer, whereas 2 eq of ***DBCO-pSar_119_*** were needed for ***T-2N_3_*** formulations to reduce cell uptake (Table 2).

**Figure 3:**
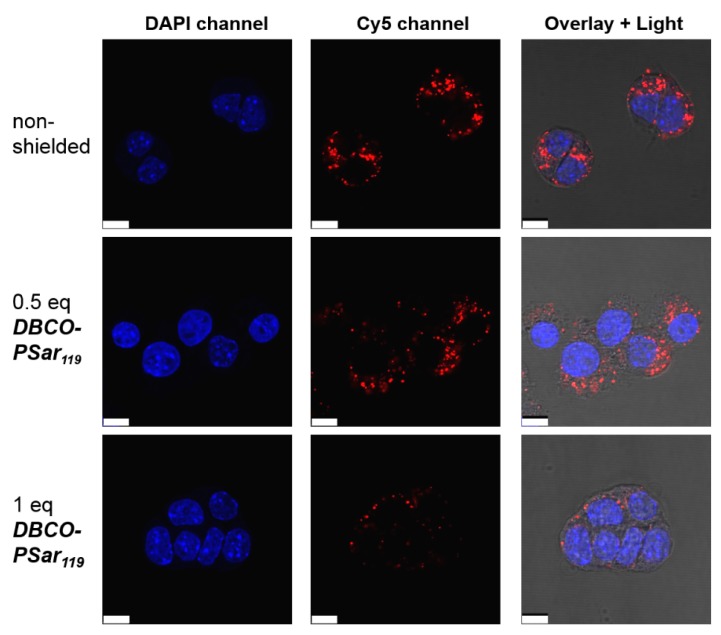
Intracellular distribution of ***T-1N_3_*** siRNA formulations in Neuro2a-eGFP-Luc cells with increasing equivalents (eq mol/mol) of ***DBCO-pSar_119_*** acquired with a confocal laser scanning microscop. Cells were incubated with the formulations for 4 h and washed with PBS buffer. siRNA was spiked with 20% Cy5-labeled siRNA (red), nuclei were stained with DAPI (blue). The overlay image shows the light microscope image and the merged DAPI and Cy5 channels. Scale bar: 10 µm.

The effect on internalization can be visualized by confocal laser scanning microscopy (CLSM). Cells were incubated with ***T-1N_3_*** siRNA formulations for 4 h and the Cy5-labeled siRNA (red) representing the localization of the polyplex was detected (Figure 3). Compared to the unshielded material that was avidly taken up by cells, 0.5 eq of ***DBCO-pSar_119_*** showed a slight reduction in cellular internalization. For 1 eq of ***DBCO-pSar_119_***, only a few polyplexes were taken up by cells, indicating efficient shielding ability. This experiment reconfirmed the observations already made in the flow cytometry studies.

In conclusion, covalent surface modification of polyplexes by SPAAC reduced cell binding and uptake substantially. Interestingly, the increase of azide functionalities in the ***T-2N_3_*** backbone did not lead to a better surface passivation of the formed polyplex. As depicted in Table 2, both polyplexes behave comparably and differ only slightly at full polysarcosinylation levels. This observation may relate to differences in microstructure between ***T-1N_3_*** and ***T-2N_3_*** based polyplexes, which seem to influence the accessibility of azide on the polyplex surface.

### 3.5. Distribution of pSar-Functionalized Polyplexes In Vivo

After the shielding ability of pSar-functionalized polyplexes could be demonstrated in biophysical and in vitro assays, we aim to explore the in vivo behavior of polysarcosinylated polyplexes. For in vivo biodistribution studies, the unshielded ***T-1N_3_*** siRNA polyplex, which showed the lowest interaction with cells, was used to prepare a formulation to which either ***DBCO-PEG5k*** and a formulation using acetylated polysarcosine (***DBCO-pSar_119_-Ac***; Figure 1, bottom) was covalently linked by SPAAC. The acetyl end group of ***DBCO-pSar_119_-Ac*** seems to be better comparable to the commercial methoxylated PEG agent in terms of surface polarity. The cap of the N-terminal sarcosine slightly reduced migration distance of polyplexes in the gel in comparison to the non-acetylated pSar (Table S2, Figure S4). ***T-1N_3_*** siRNA polyplexes were prepared with 50% Cy7-labeled siRNA followed by 4 h incubation with 1 eq of the respective shielding agent per oligomer. The final concentration of siRNA in the formulation was 200 ng/µL in this experiment. 50 µg of siRNA and oligomers at an *N*/*P* ratio of 10 were used.

**Figure 4:**
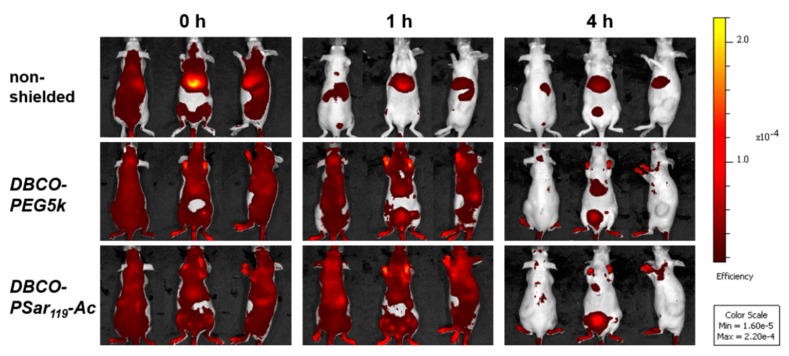
Biodistribution of ***T-1N_3_*** siRNA formulations (50 μg siRNA; 50% Cy7-labeled) in NMRI-nude mice bearing Neuro2a tumors after i.v. administration. NIR fluorescence bioimages show formulations with 1 eq ***DBCO-PEG5k***, 1 eq acetylated ***DBCO-pSar_119_***-***Ac*** or HBG buffer (non-shielded). Experiments were performed with two mice per group for time points until 60 min and one mouse per group for later time points; a representative mouse of each group is shown. Mice are presented in the dorsal, ventral and lateral view.

The formulations were administered to Neuro2a tumor-bearing mice via tail-vain injection and the distribution of the near infrared (NIR) fluorescent dye attached to the siRNA was monitored at various time points over 24 h by bioimaging in mice (Figure 4 and Figure S5). The unshielded polyplexes started accumulating in the liver after 15 min. Such a finding could also be observed for other unshielded siRNA formulations with cationic T-shape structures in previous work [89,90]. In contrast to the unshielded polyplexes, both shielded formulations showed much-extended circulation times and tumor accumulation. 60 min after i.v. injection of the material, the shielded formulations were still detectable in all parts of the body including the tumor site. The intensity of the signals decreased after 4 h, indicating a slow removal of polyplexes from circulation. The strongest signals remained in liver and bladder, which indicates that the stability of polyplexes coated with PEG or pSar is enhanced but not infinite and the labeled siRNA can leave the polyplex ending up in liver and kidney. To further enhance stability bioreversible core cross-linking is required [79,80,97]. In mice injected with shielded polyplexes, a strong signal was detected in the exposed periphery, such as the paws after more than 4 h (Figure S5). In direct comparison, polyplexes with the ***DBCO-pSar_119_-Ac*** and the ***DBCO-PEG5k*** displayed negligible differences in biodistribution, circulation time or tumor accumulation. A pronounced accumulation at the tumor site, however, could not be observed and may require further strategies to enhance tumor accessibility and retention.

### 3.6. Attachment of the Targeting Ligand Folate to Polysarcosine

**Figure 5:**
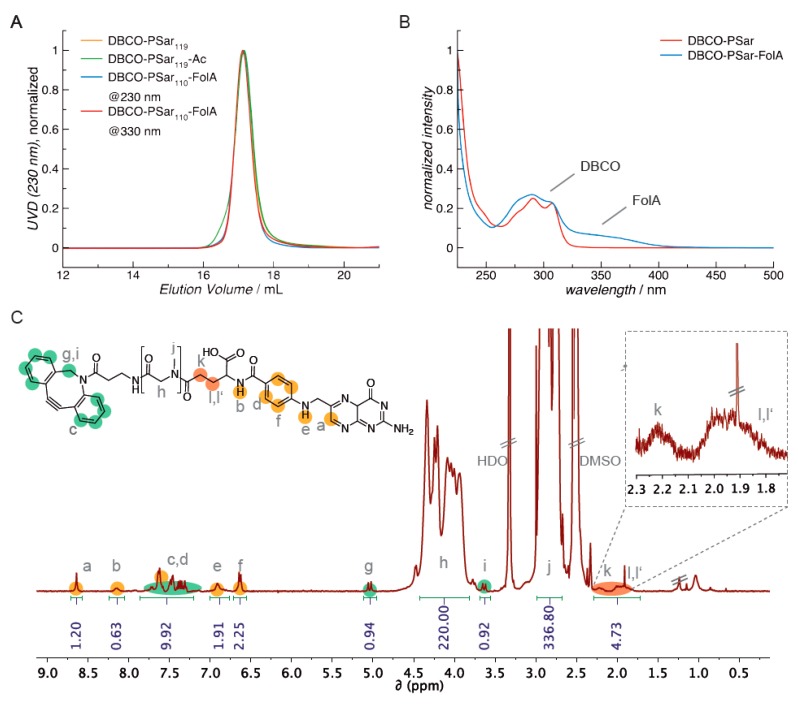
Characterization of DBCO-pSar ligands for post-shielding of polyplexes. (**A**) GPC elugrams of DBCO-pSar in HFIP with different end groups; (**B**) UV-vis spectrum of DBCO-pSar and DBCO-pSar-FolA, respectively; (**C**) ^1^H-NMR spectrum of DBCO-pSar-FolA in DMSO-*d6* (400 MHz).

The inhibition of unspecific cell association is an important requirement for any specific interaction with cell surface receptors or proteins in solution. Thus, attaching a targeting ligand onto shielded polyplexes is expected to enable targeting of specific cell surface receptors. Since pSar can be further functionalized at its free secondary amino function, we chose folic acid (FolA) as a ligand to be conjugated to the *N*-terminus of ***DBCO-pSar_110_*** by peptide bond formation. Folic acid is an interesting ligand, because it is a commercially available small molecule with carboxyl groups for conjugation and it is the natural ligand to the folic acid receptor (FR) overexpressed on several tumor types, e.g., prostate cancer [82,83,84,98,99,100,101]. The applied coupling conditions using equimolar amounts of folic acid, DBCO-pSar polymer and coupling reagents (2-(1*H*-benzotriazole-1-yl)-1,1,3,3-tetramethyluronium hexafluorophosphate (HBTU), 1-hydroxybenzotriazole (HOBt)) and the steric hindrance of the polymer avoid the formation of divalent folic acid conjugates. Concerning regioselectivity, it has been reported that both isoforms result for *N*,*N*-dicyclohexylcarbodiimide (DCC)-mediated amidation in DMSO or DMSO/DMF, but with an observed regioselectivity of 80% for the γ-conjugate (***DBCO-pSar_110_-FolA***; Figure 1 and Figure 5, Table S1) [102]. 

The properties of ***T-1N_3_*** siRNA polyplexes equipped with this negatively charged ligand changed in an unexpected way. For increasing equivalents, aggregates with high polydispersity were found by DLS (Table S1). An interesting observation was the change in size from around 80 to 25 nm of nanoparticles when folic acid was involved, which indicates strong compactation of the polyion complex. For small degrees of particle surface modification with ***DBCO-pSar_110_-FolA***, the size of polyplexes only changed marginal compared to unshielded polyplexes.

For higher equivalents, aggregates were found in DLS measurements. Similar findings were observed for folic acid-targeted lipopolyplexes before [103]. In the latter case, aggregation could be prevented by incorporation of tetra-glutamylated folic acid into the agent. We observed that for further increase of ***DBCO-pSar_110_-FolA*** to equimolar ratios, small defined particles of ~25 nm were found. This can be explained with ***DBCO-pSar_110_-FolA***-induced instability following a complete rearrangement of particles to a uniform population. The folic acid’s chemical properties—its hydrophobic character and negative charge—seem to play a major role in this reassembly process, because it was not observed for untargeted polysarcosine-shielded particles. We need to state clearly that currently the effect of FolA on polyplex formation is not understood and requires more detailed physicochemical studies.

When testing the folate targeted formulation with ***DBCO-pSar_110_-FolA*** on a FR-overexpressing KB/eGFPLuc cell line, we found that targeted polyplexes showed increased binding to the cell surface, which could be blocked by folic acid competition (Figure S6A) ensuring FR mediated binding. Much to our surprise, the internalization of the polyplexes into the cell was extremely low (Figure S6A,B). As a consequence, no gene silencing activity was achieved with such systems (Figure S6C). Limitations in endosomal escape, often reported as being responsible for bad transfection efficiencies [104,105,106,107,108], can be excluded, since co-incubation with the lysosomotropic agent chloroquine did not improve gene silencing activity (Figure S6D). The trafficking of the vitamin folate via FR is reported to occur by a non-caveolar, non-clathrin pathway also known as CLIC/GEEC endocytosis pathway [105,109]. For folate-targeted nanoparticles however, it could be demonstrated that pathways like caveolae- and clathrin-mediated endocytosis occur [106,107,108]. The size and the ligand density on the particle surface were reported to influence the cellular uptake pathway. The well shielded, ~25 nm siRNA polyplexes do not seem to trigger any of the pathways in HeLa-derived KB cells efficiently. Further, the bioreducible carrier ***T-1N_3_*** within the liopolyplex might be an easy prey for disulfide cleavage, which was reported to occur distinctly in the extracellular environment of HeLa cells [110]. The consequence of insufficient cellular uptake was a lack of gene silencing activity. This effect has been observed for folate-targeted polyplexes with 3.5 kDa PEG chains before [86]. At this point, we cannot provide an explanation for the observed findings and further studies need to be conducted to understand the fact that specific receptor binding was achieved, while receptor mediated endocytosis seems to be inhibited. In light of these in vitro data, a transfer of targeted polyplexes to in vivo studies was not performed based on ethical considerations.

## 4. Conclusions

To investigate the use of ability of pSar to shield polyplexes and enhance their circulation times and reduce unspecific interactions, we synthesized a polyplex formulation based on sequence defined lipo-oligomers and applied PEG and pSar based polymers for shielding of the preformed polyplexes. In previous work, a novel class of redox-sensitive lipo-oligomers for siRNA delivery was successfully established [39]. One of the best performing candidates from this work was chosen and extended by a click-reactive azide functionality, resulting in carrier ***T-1N_3_***. After the formation of siRNA polyplexes, the particle surface was functionalized with the shielding agent polysarcosine. The SPAAC could be performed between the DBCO-pSar polymer and the azide-containing lipo-oligomers within the polyplex. In addition, it was demonstrated that the grafting could be controlled stoichiometrically introducing a shielding layer. The shielding of the formed pSar corona has been observed in vitro in gel retardation assays and cell studies. In contrast to unmodified polyplexes, binding and cellular uptake was substantially reduced for all pSar-modified systems.

Furthermore, biodistribution in mice revealed that 8 kDa polysarcosine can clearly expand the circulation of the siRNA lipopolyplexes from several minutes to hours. The difference in non-shielded and shielded formulations is most pronounced at the 60 min time point, where in case of non-shielded polyplexes, most of the polyplexes have accumulated in the liver, but stable circulation is still observed for pSar-shielded polyplexes. While the biodistribution between non-modified polyplexes and polysarcosinylated systems differ substantially, such systems behaved similar to PEGylated polyplexes in vivo. Therefore, we can conclude that in terms of polyplex shielding, pSar and PEG behave identically and can be both applied to reduce unspecific interactions of lipo-oligomer polyplexes and thus enhance blood circulation substantially from minutes to hours. When DBCO-pSar was, however, modified with folic acid to target cell surface receptors, not only the size of polyplexes was reduced from 80 to 25 nm, but also specific binding to FR-positive KB cell membranes did not boost cellular internalization. Therefore, we need to conclude that further investigations are necessary to combine favorable in vivo shielding with efficient receptor-targeted gene silencing for pSar-functionalized lipo-oligomer polyplexes.

## Figures and Tables

**Table 1:** Particle size (z-average) and zeta potential of pSar-shielded siRNA formulations determined by a dynamic light scattering (DLS) zetasizer. siRNA polyplexes were prepared at *N*/*P* 12.

siRNA Formulation	eq *DBCO-pSar_119_*	z-Average (nm)	PDI	Mean Zeta Potential (mV)
*T-1N_3_*	0	81.0 ± 5.0	0.26 ± 0.02	20.9 ± 0.9
	0.5	86.7 ± 2.8	0.24 ± 0.02	9.4 ± 0.5
	1	91.8 ± 2.9	0.26 ± 0.02	8.5 ± 0.6
	2	96.8 ± 4.0	0.27 ± 0.02	6.0 ± 1.1
*T-2N_3_*	0	90.6 ± 0.9	0.15 ± 0.03	17.2 ± 0.8
	0.5	98.7 ± 1.3	0.15 ± 0.01	7.7 ± 0.6
	1	102.3 ± 2.1	0.19 ± 0.01	6.3 ± 1.0
	2	105.1 ± 1.9	0.17 ± 0.01	2.5 ± 0.3

**Table 2:** Mean fluorescence intensity (MFI) of Cy5-labeled siRNA formulations (top: ***T-1N_3_***; bottom: ***T-2N_3_***) shielded with increasing equivalents (eq mol/mol) of ***DBCO-pSar_119_*** for cellular internalization determined by flow cytometry.

siRNA Formulation	eq *DBCO-pSar_119_*	MFI
*T-1N_3_*	0	881.5 ± 25.5
	0.25	780.5 ± 2.5
	0.5	715.0 ± 24.0
	1	359.0 ± 14.0
	2	245.5 ± 8.0
*T-2N_3_*	0	883.0 ± 86.0
	0.25	870.5 ± 62.5
	0.5	785.5 ± 38.5
	1	602.5 ± 37.5
	2	263.5 ± 4.5
untreated cells		2.4 ± 0.2

## Supplementary Materials

The following are available online at http://www.mdpi.com/2073-4360/10/6/689/s1, Figure S1: Synthesis of heterotelechelic DBCO-pSar polyplex shielding agents by NCA polymerization and subsequent amidation for further introduction of functionalities, Figure S2: siRNA binding ability of T-shape structures analyzed with an agarose gel shift assay, Figure S3: Cellular internalization of siRNA formulations shielded with increasing equivalents of *DBCO-pSar_119_* determined by flow cytometry, Figure S4: Electrophoretic mobility of formulations, Figure S5: Biodistribution of siRNA formulations in NMRI-nude mice bearing Neuro2A tumors after i.v. administration, Figure S6: Cellular binding, cellular internalization and transfection efficiency of folate targeted siRNA polyplexes and untargeted analogues, Table S1: Analytical data of synthesized heterotelechelic DBCO-pSar ligands, Table S2: Particle size and zeta potential of siRNA formulations determined with a DLS zetasizer.
