# Supplementary material for: Efficient Shielding of Polyplexes Using Heterotelechelic Polysarcosines
Source: Polymers (Basel). 2018 Jun 20;10(6):689. doi: 10.3390/polym10060689 (PMC6404158; doi:10.3390/polym10060689)
Supplement: Supplementary file 1 [file polymers-10-00689-s001.pdf]

Supplementary Materials: Efficient Shielding of Polyplexes Using Heterotelechelic Polysarcosines

Philipp Michael Klein, Kristina Klinker, Wei Zhang, Sarah Kern, Eva Kessel, Ernst Wagner and Matthias Barz

Table of Contents

1 **Experimental Section** 2

1.1 Syntheses of lipo-cationic compounds *T-1N3* (*1073*) and *T-2N3* (*1086*) 2

1.1.1 Loading of a 2-chlorotrityl chloride resin with an Fmoc protected amino acid 2

1.1.2 Solid phase syntheses of oligomers *T-1N3* (*1073*) and *T-2N3* (*1086*) 2

1.1.3 Cleavage and purification of oligomers 3

1.2 Proton ^{1}H-NMR spectroscopy 3

1.3 MALDI mass spectrometry 3

1.4 Statistical analysis 3

2 **Supporting Figures** 4

3 **Supporting Tables** 8

4 **Analytical data** 9

4.1 ^{1}H-NMR spectra of oligomers 9

4.2 ^{1}H-NMR spectra of DBCO agents 10

4.3 Mass spectra of oligomers 11

4.3.1 Full mass spectra of oligomers 11

5 **References** 12

1. Experimental section

1.1. Syntheses of lipo-cationic compounds T-1N$_3$ (1073) and T-2N$_3$ (1086)

1.1.1 Loading of a 2-chlorotrityl chloride resin with an Fmoc protected amino acid

After swelling of 750 mg of a 2-chlorotrityl chloride resin (1.55 mmol chloride/g) in water-free DCM for 10 min, 0.3 equivalents (equiv.) of the first Fmoc protected amino acid (Fmoc-Tyr(tBu)-OH or Fmoc-Lys(N$_3$)-OH) and 0.3 equiv. DIPEA per chloride resin were added to the resin for 1 h. The reaction solvent was drained and a mixture of DCM/MeOH/DIPEA (80/15/5) was added twice for 10 min. After the removal of the reaction mixture, the resin was washed 5 times with DCM.

About 30 mg of the resin were removed and dried to determine the loading of the resin. Therefore, an exact amount of resin was treated with 1 mL deprotection solution (20% piperidine in DMF) for 1 h. The solution was diluted and absorption was measured at 301 nm. The loading was then calculated according to the equation: resin load [mmol/g] = (A × 1000)/(m(mg) × 7800 × df) with df as dilution factor.

The resin was treated twice with 20% piperidine in DMF and twice with 20% piperidine DMF with 2% DBU to remove the fluorenylmethoxycarbonyl Fmoc protection group. Reaction progress was monitored by Kaiser test [1]. Afterwards, the resin was washed with DMF, DCM and *n*-hexane and dried in vacuo.

1.1.2 Solid phase syntheses of oligomers ***T-1N$_3$* (*1073*)** and ***T-2N$_3$* (*1086*)**

The artificial amino acid Fmoc-Stp(Boc)$_3$-OH was synthesized according to the protocol published by Schaffert et al. [2], and Fmoc-succinoyl-cystamine according the protocol of Klein et al. [3]. Oligoamides were synthesized using a 2-chlorotrityl resin preloaded with the first C-terminal amino acid (see 0). The sequence (C → N) [Y(tBu)]$_3$-[Stp(Boc)$_3$]$_2$-K(Dde)-[Stp(Boc)$_3$]$_2$-[Y(tBu)]$_3$ for ***T-1N$_3$* (*1073*)** and K(N$_3$)-[Y(tBu)]$_3$-[Stp(Boc)$_3$]$_2$-K(Dde)-[Stp(Boc)$_3$]$_2$-[Y(tBu)]$_3$ for ***T-2N$_3$* (*1086*)** was synthesized with a SyroWave™ synthesizer (Biotage, Uppsala, Sweden). Coupling steps were carried out using 4 eq Fmoc-amino acid, 4 eq HOBt, 4 eq HBTU, and 8 eq DIPEA in NMP/DMF (5 mL g^{-1} resin) twice for 12 min at 50 °C. Equivalents were calculated relative to free resin-bound amines (1 eq). Fmoc deprotection was accomplished by 5 × 10 min incubation with 20% piperidine in DMF (7 mL g^{-1} resin). Washing was accomplished by 6 × 1 min DMF (8 mL g^{-1} resin) after each coupling and deprotection step. The remaining coupling steps were performed manually under standard Fmoc solid-phase peptide synthesis conditions using syringe microreactors. Coupling steps were carried out using 4 eq Fmoc-amino acid, 4 eq HOBt, 4 eq PyBOP, and 8 eq DIPEA in DCM/DMF (50/50) (10 mL g^{-1} resin) for 90 min. Fmoc deprotection was accomplished by 4 × 10 min incubation with 20% piperidine in DMF (10 mL g^{-1} resin). A washing procedure comprising 3 × 1 min DMF, 3 × 1 min DCM incubation (10 mL g^{-1} resin) and a Kaiser test were performed after each coupling and deprotection step. In case of a positive result of the Kaiser test after coupling, the last coupling step was repeated. In case of a negative result after deprotection, the last deprotection step was repeated. Fmoc-Lys(N$_3$)-OH was coupled to the backbone and after the removal of the Fmoc protecting group, the N-terminal NH$_2$-group was protected with 10 eq Boc anhydride and 10 eq DIPEA in DCM/DMF. Dde-deprotection was performed 20 times with the automatic synthesizer. A hydrazine/DMF solution (2/98) was added and vortexed for 2 min. The reaction solvent was drained and fresh solution was added again. Afterwards, the resin was washed with 5 × 1 min DMF, 5 × 1 min 10% DIPEA in DMF and 3 × 1 min DCM (10 mL g^{-1} resin). After the coupling of Fmoc-Gly-OH, Fmoc-succinoyl-cystamine was coupled without HOBt and only DMF was used as solvent (Kaiser tests are not always correct after the deprotection). All couplings after Fmoc-succinoyl-cystamine were carried out without HOBt. Symmetrical branching was introduced using Fmoc-Lys(Fmoc)-OH. In the final step 5β cholanic acid was coupled.

1.1.3 Cleavage and purification of oligomers

The cleavage of the structures off the resin were performed by incubation with TFA/TIS/H_2O (95/2.5/2.5) (10 mL g^{-1} resin cooled to 4 °C prior to addition) for 60 min followed by immediate precipitation in 40 mL of pre-cooled MTBE/*n*-hexane (50/50). The oligomers were then purified by size exclusion chromatography using a Äkta purifier system (GE Healthcare Bio-Sciences AB, Uppsala, Sweden), a Sephadex G-10 column (60 cm) and 10 mM hydrochloric acid solution/acetonitrile (70/30) as solvent. The oligomers were lyophilized.

1.2 Proton ^{1}H-NMR spectroscopy

^{1}H-NMR spectra were recorded using an AVANCE III HD 500 (500 MHz) by Bruker (Billerica, MA, USA) equipped with a 5 mm CPPBBO probe in D_2O or a Bruker AV 400 spectrometer in DMSO-*d6* (Deutero GmbH, Kastellaun, Germany) at room temperature. All spectra were recorded without TMS as internal standard and therefore all signals were calibrated to the residual proton signal of the solvent. Chemical shifts are reported in ppm and refer to the solvent as internal standard (D_2O; δ=4.79 ppm and DMSO-*d6*; δ=2.50 ppm). Integration was performed manually. The spectra were analyzed using MestreNova (Ver. 10.0 by MestReLab Research, Santiago de Compostela, Spain).

1.3 MALDI mass spectrometry

One µL matrix droplet consisting of a saturated solution of Super-DHB (sum of 2,5-dihydroxybenzoic acid and 2-hydroxy-5-methoxybenzoic acid) in acetonitrile:water (1:1) containing 0.1% (v/v) TFA was spotted on an MTP AnchorChip (Bruker Daltonics, Bremen, Germany). After the Super-DHB matrix had crystallized, one µL of the sample solution (10 mg/mL in water) was added to the matrix spot. Samples were analyzed using an Autoflex II mass spectrometer (Bruker Daltonics). All spectra were recorded in positive mode.

1.4 Statistical analysis

The results are presented as mean values of experiments performed in at least triplicates. Unless otherwise stated error bars show standard deviation (SD). In case of *in vivo* data statistical analysis of the results are presented as mean ± SEM. was evaluated by unpaired t test: *$p<0.05$; **$p<0.01$; ***$p<0.001$. Two-tailed Student's t-test, calculations and graphical presentation were performed with Prism 6 (GraphPad Software Inc., La Jolla, CA, USA) and Microsoft Excel 2013 (Microsoft Corp., Redmond, WA, USA).

2. Supporting Figures

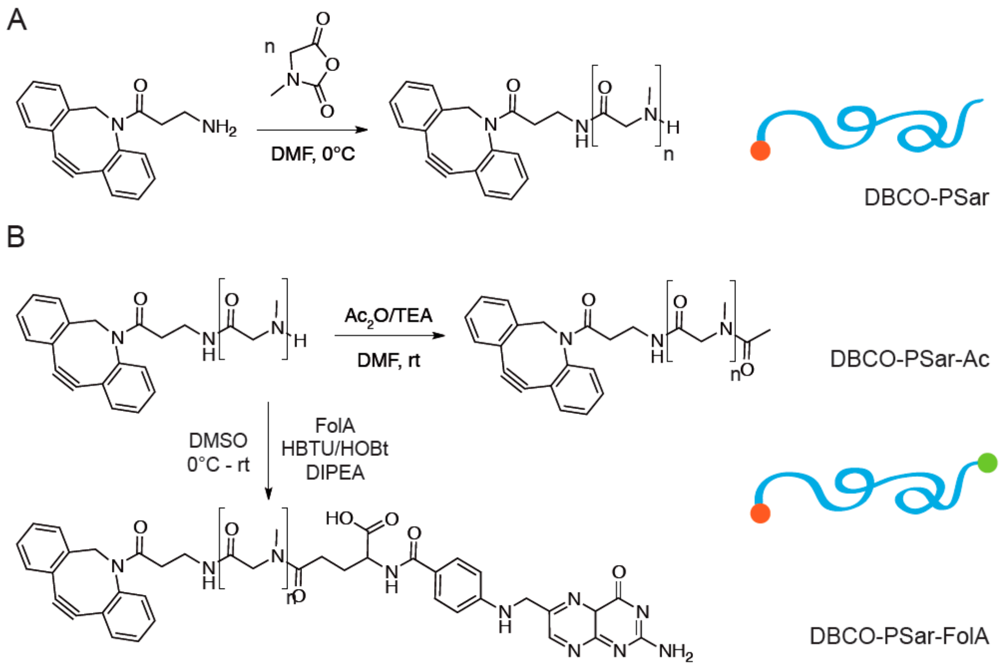

Figure S1. Synthesis of heterotelechelic DBCO-pSar polyplex shielding agents by NCA polymerization (A) and subsequent amidation for further introduction of functionalities (B).

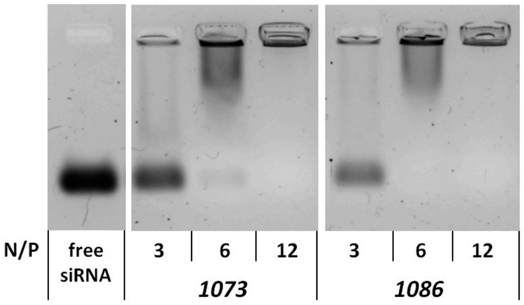

Figure S2. siRNA binding ability of T-shape structures ***T-1N3*** (***1073***) and ***T-2N3*** (***1086***) analyzed with an agarose gel shift assay. The left lane shows the running distance of free siRNA in HBG that is not complexed by lipo-oligomers. Polyplexes were tested for siRNA binding ability at different N/P ratios. 2.5% agarose gel, 100V, 40 min runtime.

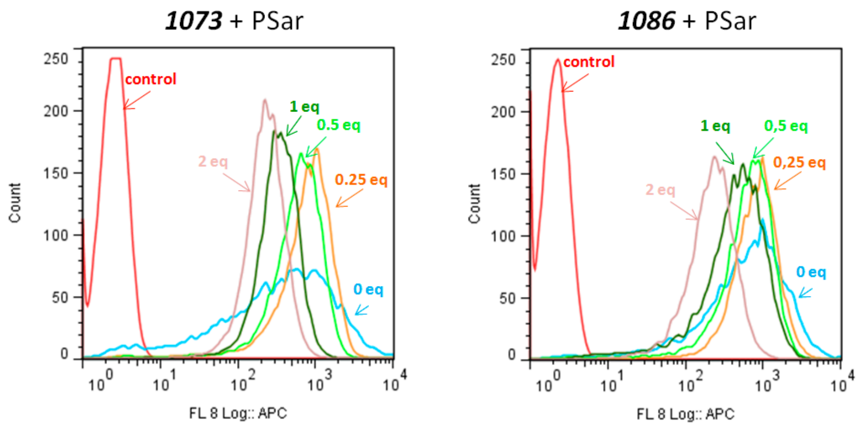

Figure S3. Cellular internalization of siRNA formulations (left: ***T-1N***$_3$ (***1073***); right: ***T-2N***$_3$ (***1086***)) shielded with increasing equivalents (eq mol/mol) of ***DBCO-pSar***$_{119}$ (pSar) determined by flow cytometry. All polyplexes were incubated for 40 min at N/P 12 with siRNA (20% Cy5-labeled), followed by ***DBCO-pSar***$_{119}$ (pSar) for 16 h at room temperature. Cells were incubated with the formulations for 4 h at 37°C and washed with PBS buffer and heparin solution. The intensity of the Cy5 signal indicates the amount of polyplexes being internalized by Neuro2A-eGFP-Luc cells. Control: HBG buffer treated cells.

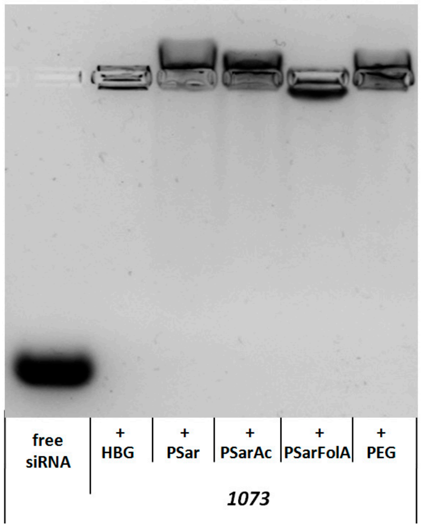

Figure S4. Electrophoretic mobility of ***T-1N***$_3$ (***1073***) siRNA formulations with 1 eq ***DBCO-pSar***$_{119}$ (pSar), 1 eq ***DBCO-pSar***$_{119}$***-Ac*** (pSarAc), 1 eq ***DBCO-pSar***$_{110}$***-FolA*** (pSarFolA), 1 eq ***DBCO-PEG5k*** (PEG) or hepes buffered glucose (HBG) analyzed with an agarose gel shift assay. All polyplexes were incubated at *in vivo* concentrations (200µg/µl) at N/P 10 for 40 min, followed by HBG, PEG or pSar incubation for 4 h at room temperature. The solutions were diluted 1 : 8 with HBG and loaded onto the gel. The left lane shows the running distance of free siRNA in HBG that is not complexed by lipo-oligomers. 1% agarose gel, 70 V, 80 min runtime.

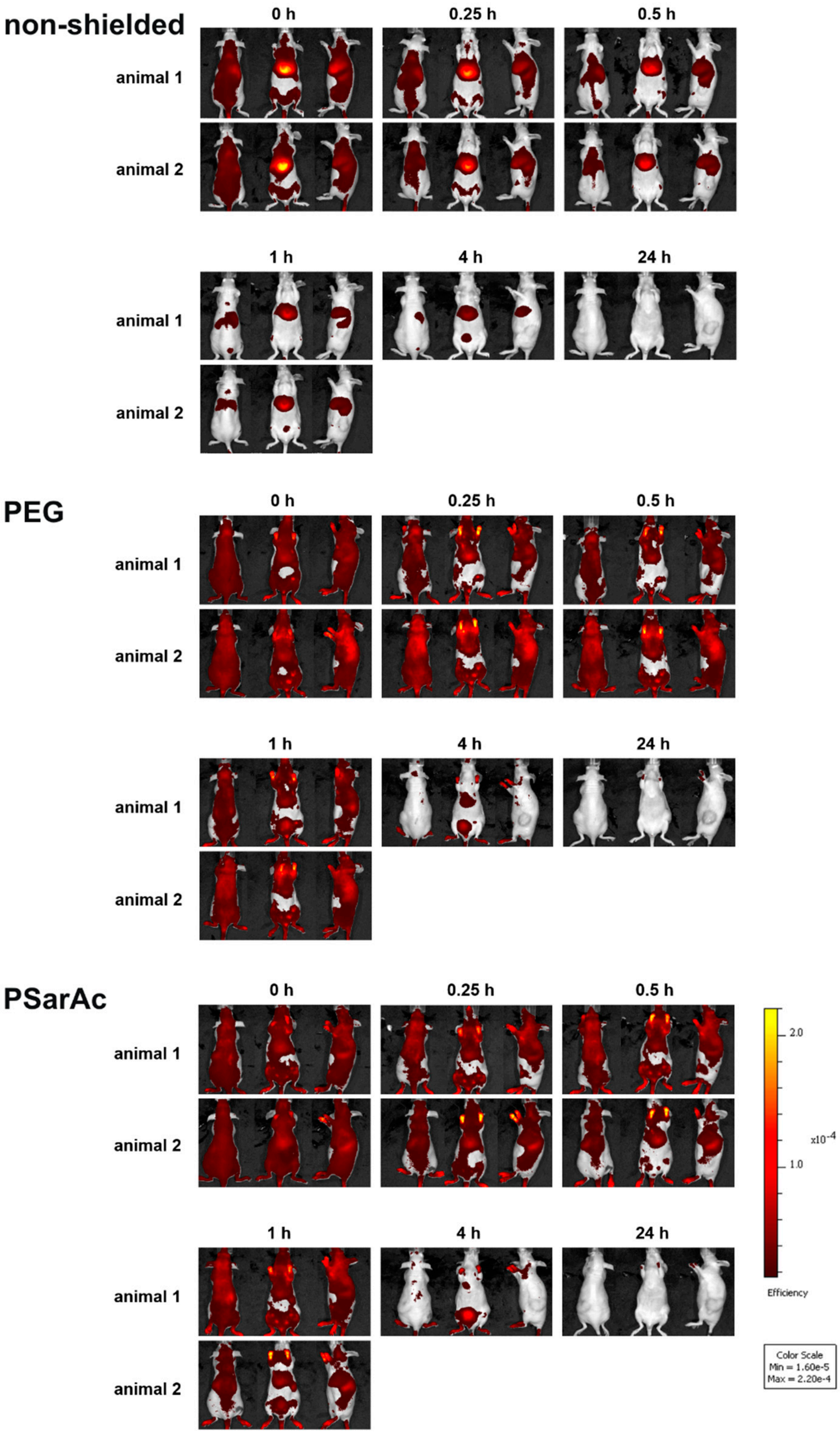

Figure S5. Biodistribution of ***T-1N₃*** (***1073***) siRNA formulations (50 µg siRNA; 50% Cy7-labeled) in NMRI-nude mice bearing Neuro2A tumors after *i.v.* administration. NIR fluorescence bioimages show formulations with 1 eq ***DBCO-PEG5k*** (PEG), 1 eq acetylated ***DBCO-pSar$_{119}$Ac*** (pSarAc) or HBG buffer (non-shielded). Experiments were performed with two animals per group for time points until 1 h and one animal per group for later time points. Animals are presented in the dorsal, ventral and lateral view.

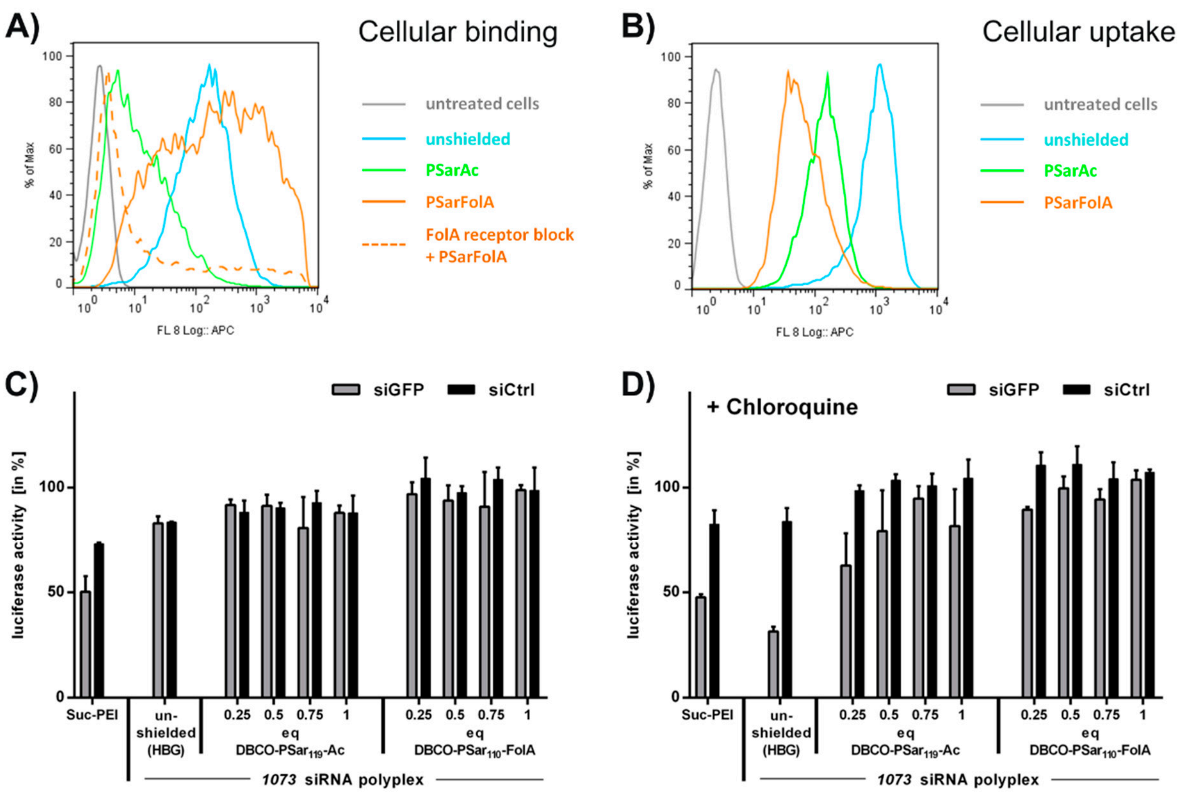

Figure S6. A) Cellular binding of Cy5-labled (20%) unshielded siRNA formulations with HBG buffer instead of agent or shielded with 1 eq (mol/mol) of ***DBCO-pSar$_{119}$-Ac*** (pSarAc), ***DBCO-pSar$_{110}$-FolA*** (pSarFolA) determined by flow cytometry. KB cells were incubated with the formulations for 30 min on ice and washed with PBS buffer. For the FolA receptor block cell culture medium with 1 mM free folate was used. B) Cellular internalization of Cy5-labled (20%) unshielded siRNA formulations or shielded with 1 eq (mol/mol) of ***DBCO-pSar$_{119}$-Ac*** (pSarAc), ***DBCO-pSar$_{110}$-FolA*** (pSarFolA) determined by flow cytometry. KB cells were incubated with the formulations for 45 min and washed with PBS buffer and heparin solution. C) Lipopolyplex formulations with eGFP-targeted siRNA (siGFP) or control siRNA (siCtrl) were tested for eGFPLuc gene silencing in KB-eGFPLuc cells. Formulations with ***T-1N$_3$*** (***1073***) siRNA polyplex modified with increasing equivalents (eq mol/mol) of ***DBCO-pSar$_{119}$-Ac***, ***DBCO-pSar$_{110}$-FolA*** or unshielded ***T-1N$_3$*** (***1073***) siRNA polyplex (addition of HBG buffer instead of agent). Cells were incubated with formulations for 45 min and fresh medium was changed; the luciferase readout was after 48 h. D) After incubation of the cells with formulations for 45 min, chloroquine was added and cells were incubated for 4 h. The medium was replaced with fresh medium and the luciferase readout was performed after 48 h. The luciferase activity of siRNA-treated cells is presented related to HBG-treated cells. Data are presented as mean value (±SD) out of triplicates. All polyplexes were incubated for 40 min at N/P 10 with siRNA, followed by shielding agent for 4 h at room temperature. Formulations were prepared at a concentration of 200 ng siRNA/µL and diluted 1 : 8 with cell culture medium before incubation with the cells.

3. Supporting Tables

Table S1. Analytical data of synthesized heterotelechelic DBCO-pSar ligands.

Agent	M/I	$X_{n(NMR)}$[a]	$M_{n(NMR)}$[a] / g mol^{-1}	$M_{n(GPC)}$[b] / g mol^{-1}	$Đ_{GPC}$
DBCO-pSar $_{119}$	100	119	8735	24819	1.12
DBCO-pSar $_{119}$***-Ac***	100	119	8735	25862	1.12
DBCO-pSar $_{110}$***-FolA***	100	110	8095	27023	1.11

[a]Determined by ^{1}H-NMR in DMSO-*d6* by relating end group signals to those of the repeating unit. [b]Relative to PMMA standards. Note that DBCO-pSar-Ac was synthesized from DBCO-pSar, a different but comparable precursor polymer (DBCO-pSar, Xn=110) was used for the synthesis of DBCO-pSar-FolA.

Table S2. Particle size (z-average) and zeta potential of siRNA formulations determined with a DLS zetasizer. Formulations were prepared at 200 ng siRNA/μL concentration with ***DBCO-pSar*** $_{119}$ (pSar), acetylated ***DBCO-pSar*** $_{119}$***Ac*** (pSarAc), ***DBCO-PEG5k*** (PEG), targeted ***DBCO-pSar*** $_{110}$***FolA*** (pSarFolA), or hepes buffered glucose (HBG) and diluted 1 : 8 with HGB before size measurements

T-1N $_3$ **(*1073*) siRNA formulation**	**N/P**	**z-average [nm]**	**Mean PDI**	**Mean Zeta Potentianl [mV]**
**+ HBG buffer**	10	161.2 ± 5.3	0.15 ± 0.01	33.3 ± 0.3
**+ 1 eq pSar**	10	173.8 ± 1.1	0.13 ± 0.02	9.9 ± 0.2
**+ 1 eq pSarAc**	10	188.6 ± 3.8	0.17 ± 0.01	0.1 ± 0.5
**+ 1 eq PEG**	10	169.9 ± 1.4	0.15 ± 0.01	3.9 ± 0.2
**+ 0.25 eq pSarFolA**	10	171.2 ± 3.9	0.22 ± 0.01	7.7 ± 0.3
**+ 0.5 eq pSarFolA**	10	1013.8 ± 195.1	0.79 ± 0.09	5.8 ± 0.2
**+ 0.75 eq pSarFolA**	10	4092.3 ± 384.4	0.66 ± 0.30	3.4 ± 0.1
**+ 1 eq pSarFolA**	10	25.4 ± 0.7	0.20 ± 0.01	1.5 ± 0.4

4. Analytical data

4.1 ^{1}H-NMR spectra of oligomers

T-1N$_3$ (***1073***): Sequence (C→N): Y$_3$-Stp$_2$-K-ε[G-ssbb-K-α,ε(CholA)$_2$]αStp$_2$-Y$_3$-K-ε(N$_3$)

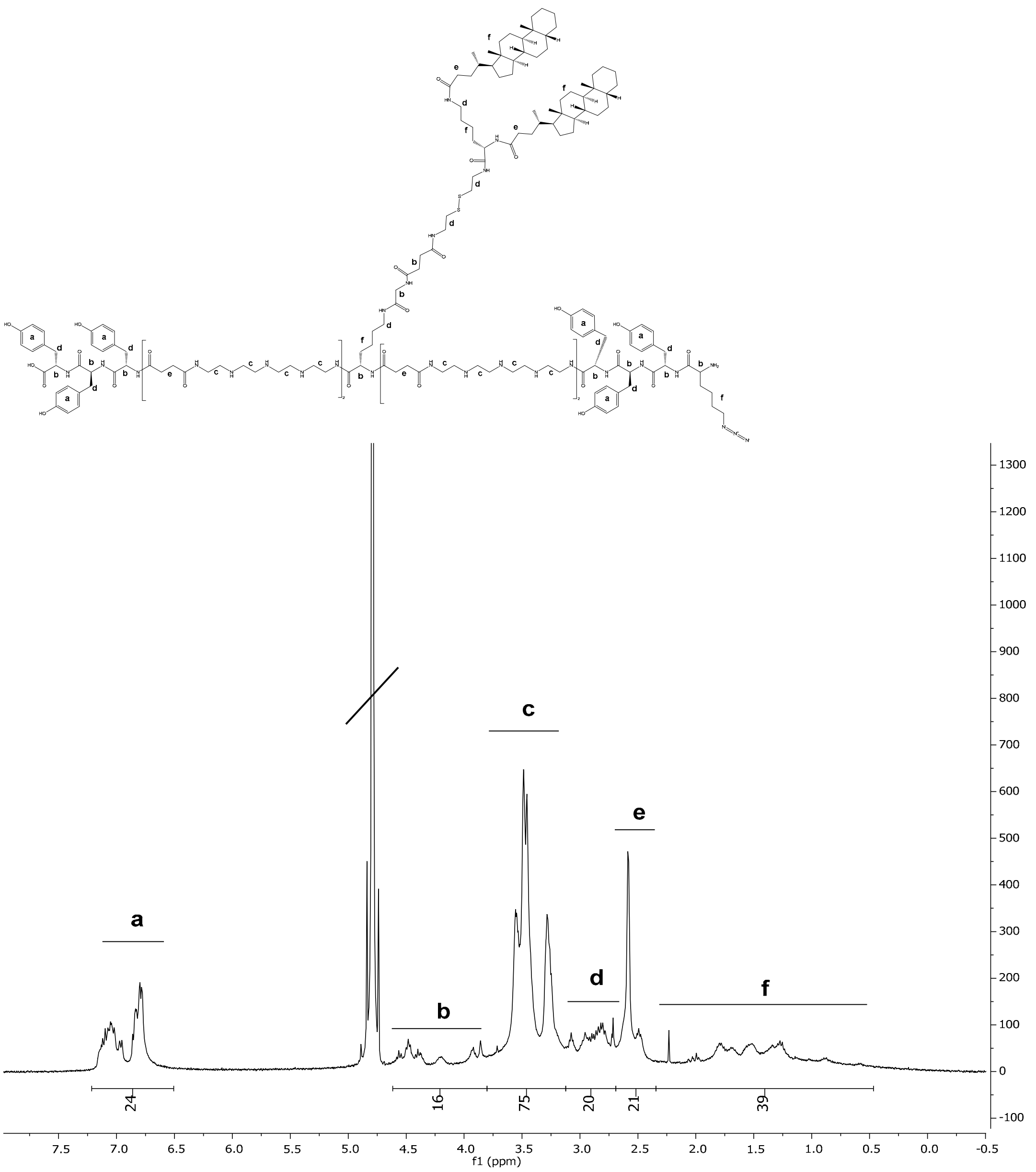

^{1}H-NMR (500 MHz, D$_2$O) δ (ppm) = 0.40-2.30 (m, 96 H, βγδH lysine, βγδεH azidolysine, cholanic acid), 2.3-2.7 (m, 24 H, -CO-CH2-CH2-CO- Stp and ssbb, -CO-CH2- cholanic acid), 2.70-3.15 (m, 24 H, εH lysine and tyrosine, -CH2- ssbb), 3.15-3.80 (m, 64 H, -CH2- Tp), 3.65-4.65 (m, 11 H, αH amino acids), 6.60-7.35 (m, 24 H, -CH- tyrosine).

***T-2N*₃ (*1086*)**: Sequence (C→N): K-ε(N_3)-Y_3-Stp_2-K-ε[G-ssbb-K-α,ε($CholA)_2$]$\alpha$$Stp_2$-$Y_3$-K-ε($N_3$)

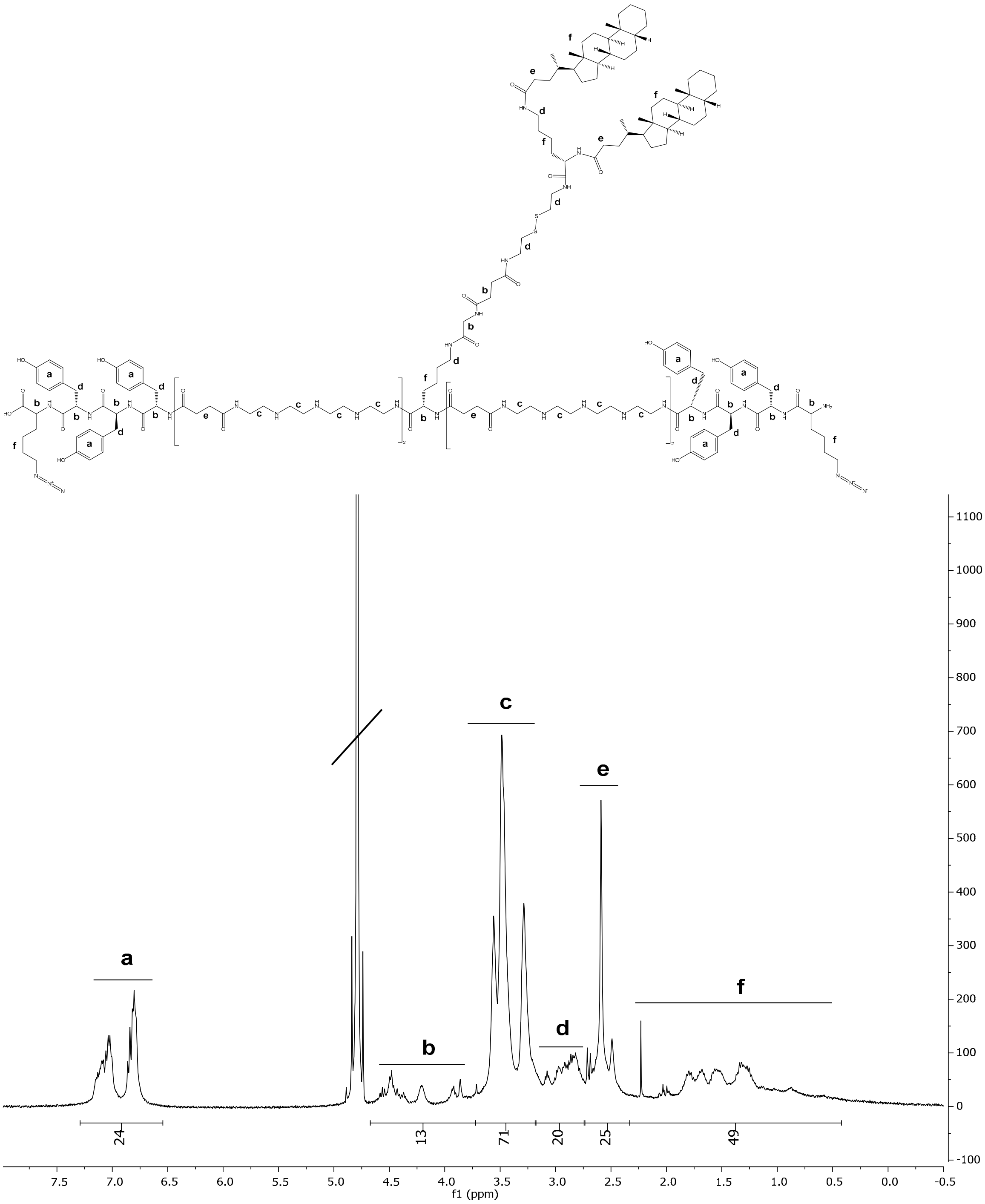

^{1}H-NMR (500 MHz, D_2O) δ (ppm) = 0.40-2.30 (m, 104 H, βγδH lysine, βγδεH azidolysine, cholanic acid), 2.3-2.7 (m, 24 H, -CO-CH2-CH2-CO- Stp and ssbb, -CO-CH2- cholanic acid), 2.70-3.15 (m, 24 H, εH lysine and tyrosine, -CH2- ssbb), 3.15-3.80 (m, 64 H, -CH2- Tp), 3.65-4.65 (m, 12 H, αH amino acids), 6.60-7.35 (m, 24 H, -CH- tyrosine).

4.2 ^{1}H-NMR spectra of DBCO agents

DBCO-pSar

Published in Klinker et al. [4]

DBCO-pSar$_{119}$-Ac

^{1}H NMR (400 MHz, DMSO-d$_6$): δ/ppm = 2.0 (d, br, 3H, -CH$_3$), 2.58–3.11 (br, 3nH, -N–CH$_3$), 3.73–4.57 (br, 2nH, -CO–CH$_2$–N) 7.86–7.10 (m, 8H, -C$_4$H$_4$ (2x)).

DBCO-pSar$_{110}$-FolA

^{1}H-NMR (400 MHz, DMSO-d$_6$) δ/ppm = 1.75–2.11 (m, br, 2H, β-CH$_2$), 2.12–2.28 (m, br, 2H, γ-CH$_2$), 3.09–2.66 (m, 3nH, -N–CH$_3$), 3.63 (d, $^3J_{H,H}$ = 14.1Hz, 1H, -N–*CH$_2$*– aryl$_{DBCO}$), 3.77–4.45 (m, 2nH, -CO–CH$_2$–N), 4.47 (d, 1H, $^3J_{H,H}$ = 6.1 Hz, -NH–*CH$_2$*), 5.04 (d, *J* = 14.0 Hz, 1H, -N–*CH$_2$*–aryl$_{DBCO}$), 6.64 (d, $^3J_{H,H}$ = 8.7 Hz, 1H, *meta*-H), 6.92 (s, 1H, aryl–N*H*–CH$_2$-), 7.15–7.77 (m, 10H, -C$_4$H$_{4(DBCO)}$ (2x); *ortho*-H), 8.16 (s, 1H, -COOH–C–N*H*–C=O), 8.65 (s, 1H, –C–C*H*–C=N-).

4.3 *Mass spectra of oligomers*

Table S2. Summarizing table oligomers. Mass data recorded with a Bruker MALDI-TOF instrument

Oligomer	Molecular formula	[M+H]$^+$ calc.	[M+H]$^+$ found
T-0N$_3$* (*992*)	$C_{172}H_{273}N_{33}O_{28}S_2$	3314.0	3314.2
***T-1N$_3$* (*1073*)**	$C_{178}H_{283}N_{37}O_{29}S_2$	3468.1	3466.2
***T-2N3* (*1086*)**	$C_{184}H_{293}N_{41}O_{30}S_2$	3622.2	3621.3

* published in Klein et al. [3]

4.3.1 Full mass spectra of oligomers

T-1N$_3$ (***1073***)

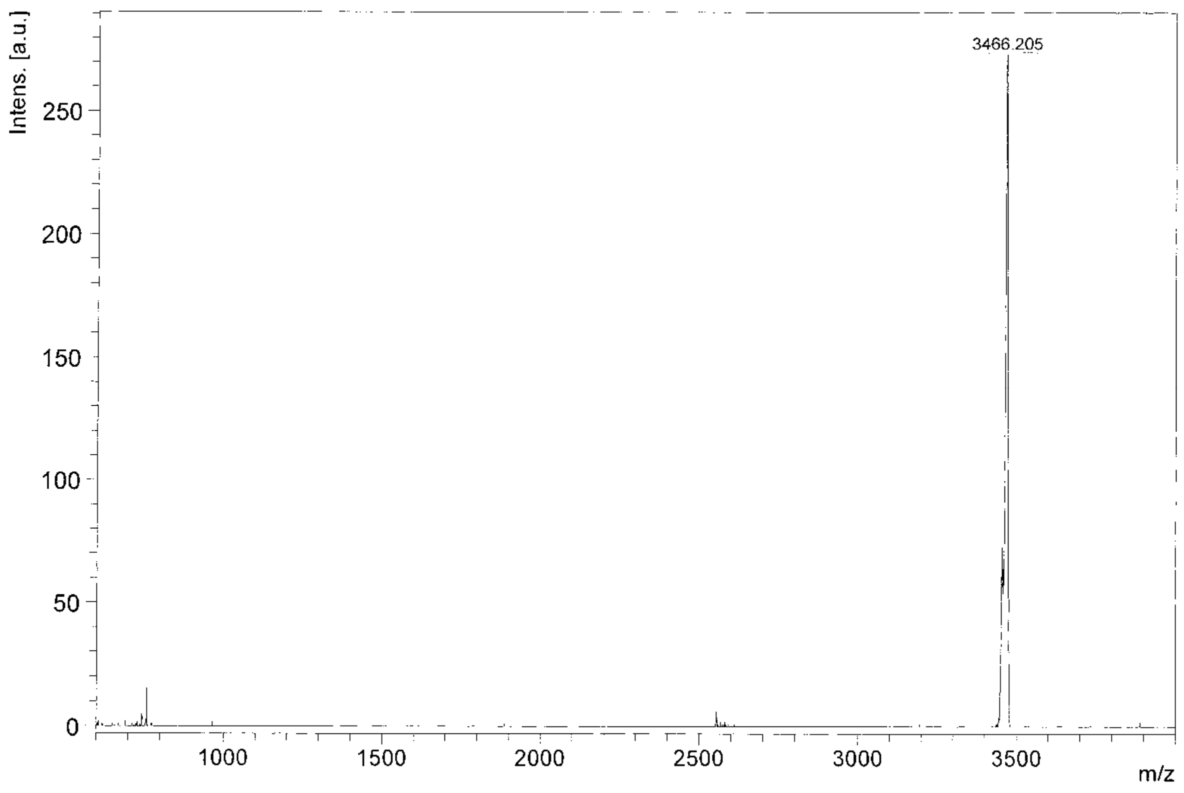

T-2N$_3$ (***1086***)

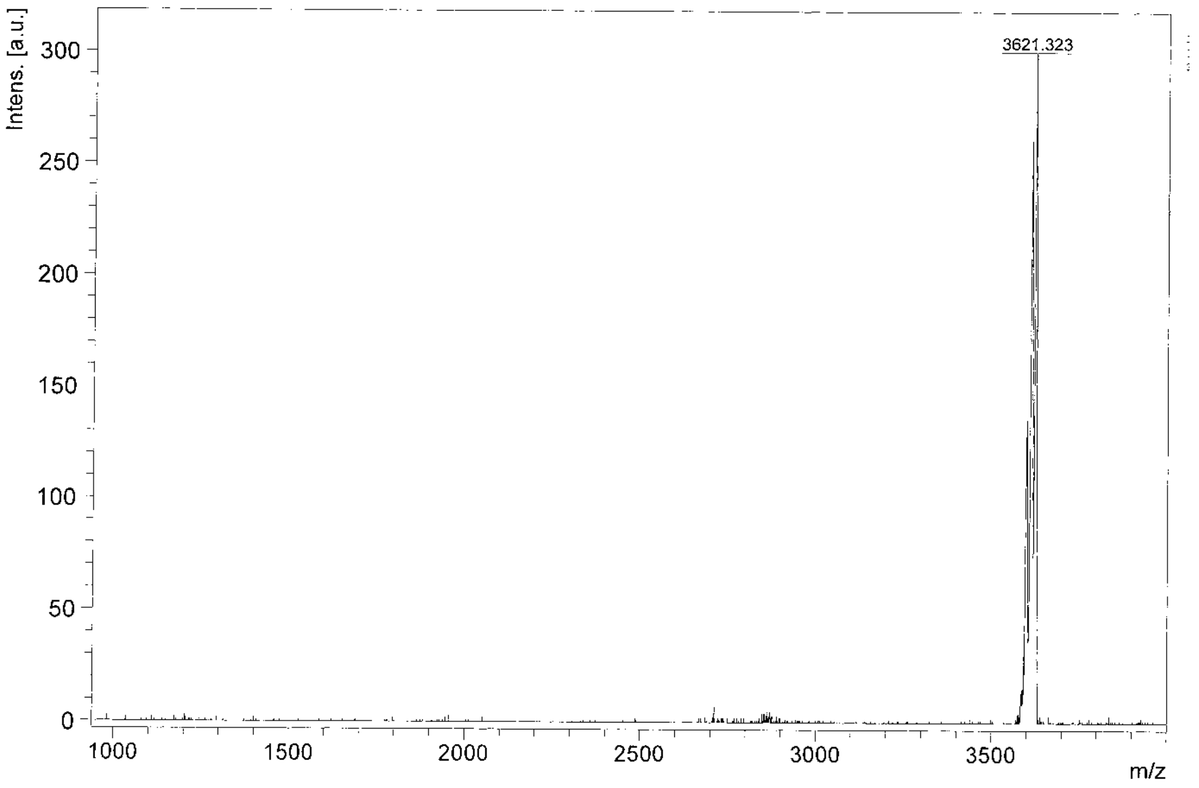

5. References

[1] E. Kaiser, R.L. Colescott, C.D. Bossinger, P.I. Cook, Color test for detection of free terminal amino groups in the solid-phase synthesis of peptides, Anal Biochem 34(2) (1970) 595-8.

[2] D. Schaffert, N. Badgujar, E. Wagner, Novel Fmoc-polyamino acids for solid-phase synthesis of defined polyamidoamines, Org Lett 13(7) (2011) 1586-9.

[3] P.M. Klein, S. Reinhard, D.J. Lee, K. Muller, D. Ponader, L. Hartmann, E. Wagner, Precise redox-sensitive cleavage sites for improved bioactivity of siRNA lipopolyplexes, Nanoscale 8(42) (2016) 18098-18104.

[4] K. Klinker, R. Holm, P. Heller, M. Barz, Evaluating chemical ligation techniques for the synthesis of block copolypeptides, polypeptoids and block copolypept(o)ides: a comparative study, Polymer Chemistry 6(25) (2015) 4612-4623.
